# A novel *FBN2* mutation cosegregates with congenital contractural arachnodactyly in a five‐generation Chinese family

**DOI:** 10.1002/ccr3.1693

**Published:** 2018-07-03

**Authors:** Shiyuan Zhou, Fengyu Wang, Yongheng Dou, Jiping Zhou, Gefang Hao, Chengqi Xu, Qing K. Wang, Haili Wang, Pengyun Wang

**Affiliations:** ^1^ Henan Provincial Research Institute for Population and Family Planning Zhengzhou China; ^2^ Key Laboratory of Birthdefects Prevention National Health and Family Planning Commission Zhengzhou China; ^3^ College of Life Science and Technology and Human Genome Research Center Huazhong University of Science and Technology Wuhan China; ^4^ Department of Clinical Laboratory Liyuan Hospital Tongji Medical Collage Huazhong University of Science and Technology Wuhan China

**Keywords:** congenital contractural arachnodactyly, exome sequencing, fibrillin‐2, mutation

## Abstract

We identified a novel heterozygous mutation (c.4177T>G and p.Cys1393Gly) in *FBN2* that cosegregated with congenital contractural arachnodactyly (CCA) in a five‐generation Chinese family. This mutation may cause the loss of the disulfide bond between Cys 1393 and Cys 1378 residues of fibrillin‐2. Our study expands the genetic profile of CCA.

## 1 INTRODUCTION

Congenital contractural arachnodactyly (CCA, OMIM #121050) is a rare congenital disorder characterized by abnormal connective tissue.^1^ CCA was first described in 1968, and it was found to share some common characteristics with Marfan syndrome (MFS), such as arachnodactyly, pectus deformities, dolichostenomelia, and kyphoscoliosis.^2^, ^3^, ^4^ However, most patients with CCA do not have the ocular and cardiovascular complications, which are the typical characteristics of MFS.^5^, ^6^

Through genetic linkage analysis and mutation screening, *FBN2* gene mutations were found to correspond to the incidence of CCA in many probands or families.^7^, ^8^, ^9^, ^10^, ^11^, ^12^, ^13^, ^14^, ^15^ To date, *FBN2* is the only known susceptibility gene of CCA.^10^, ^16^, ^17^, ^18^ The *FBN2* gene encodes fibrillin‐2, which has been demonstrated to have important roles in the structure of extracellular microfibrils in elastic fiber by providing strength and flexibility to connective tissue.^14^, ^16^, ^19^ Previous studies in mouse models showed that the knockout of *FBN2* resulted in phenotypes such as forelimbs contractures, disorganized microfibrils, and bilateral syndactyly.^14^, ^20^

In this study, we performed whole‐exome sequencing in a five‐generation Chinese family that exhibited autosomal dominant CCA, resulting in the identification of a novel heterozygous mutation (c.4177T>G, p.Cys1393Gly) of *FBN2*. This mutation cosegregated with CCA in the present pedigree and was absent in normal controls. Our study may provide valuable information for the genetic diagnosis in patients with CCA and allow for subsequent studies on CCA pathogenesis due to *FBN2* mutations.

## 2 CLINICAL REPORT

**Figure 1:**
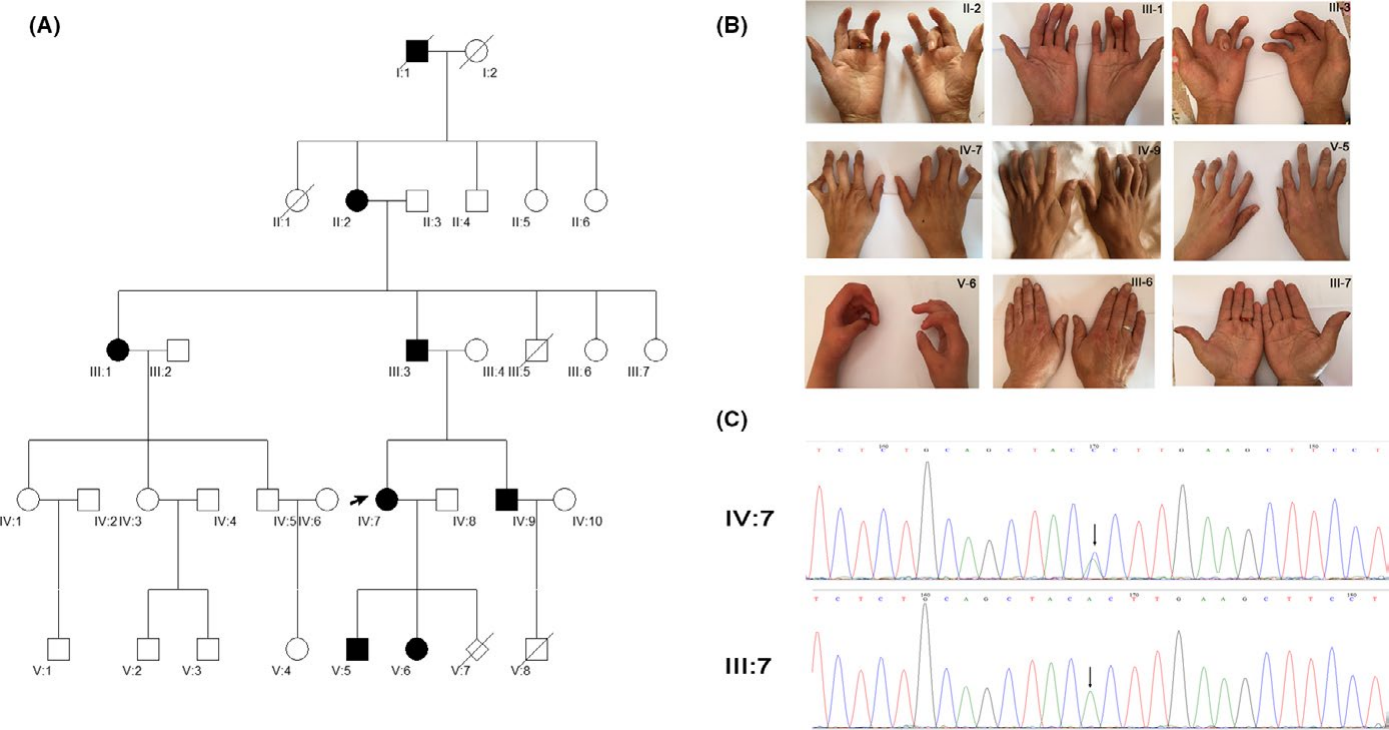
*FBN2* c.4177T>G mutation results in congenital contractural arachnodactyly (CCA) in the family. A, The affected members with CCA are depicted by a shaded black square (male) or circle (female). Generations are shown as I to III. The proband (IV:7) is indicated by an arrow, and the slash indicates deceased individuals. B, Clinical features of patients with CCA (II:2, III:1, III:3, IV:7, IV:9, V:5, and V:6) and controls (III:6 and III:7). C, Sanger sequencing to confirm the *FBN2* c.4177T>G mutation in the proband (IV:7) and the unaffected control (III:7). The mutation is marked by an arrow

The CCA pedigree in this study has 33 members across five generations, six of whom were deceased (Figure 1A). Physical tests, medical examinations, and X‐ray imaging were performed on the proband and other members of the family to determine the status of CCA. The proband (IV:7) had contractures of the fingers at birth, and the other six living members of the family showed the same phenotype (Figure 1B). Further physical examination of the 27 living members and the CCA disease revealed an autosomal dominant mode of inheritance in the present pedigree. Except for arachnodactyly and camptodactyly, all seven affected members showed no aberrant mental and motor development. No obvious aberrant results were observed from the results of blood and urine examinations. There were no signs of muscle hypoplasia, tall stature kyphoscoliosis, large joint contracture, cardiovascular abnormalities, crumpled ears, or ocular complication. Additionally, there was no heterogeneity of the phenotype between different genders.

Written informed consent was obtained from all subjects, and the study was approved by the Ethics Committees on human subject research of Huazhong University of Science and Technology (2017‐IEC‐S091). All experiments were performed in accordance with the Declaration of Helsinki.

## 3 METHODS

### 3.1 Whole‐exome sequencing

The whole‐exome sequencing was carried out by a commercial company (Kangso medical inspection, Beijing, China) following standard experimental procedures. The exome was captured using Agilent SureSelect^XT^ Human All Exon V6 Kit. The high‐throughout sequencing was based on NextSeq500 (Illumina, San Diego, CA, USA). The read length of the sequencing was paired‐end 150 bp, and the read depth was 120×. High‐throughput exome sequencing was selectively carried out on the proband (IV:7), her son (V:5), and daughter (V:6). Details of the exome sequencing are shown in Appendix S1.

### 3.2 Bioinformatics analysis of the mutations

The possible effects of the mutations on the function and structure of protein, and likelihood of pathological damage were analyzed by tools including SIFT, MutationTaster, PolyPhen‐2. Mutation Assessor, and FATHMM.

The software Rapid Automatic Detection and Alignment of Repeats (RADAR) was used to analyze the repeats of FBN2 calcium‐binding epidermal growth factor (cbEGF) motifs 18‐19.^18^

The sequences of fibrillin‐2 protein from human, mouse, chicken, zebrafish, cat, and chimpanzee were aligned. and the conservation was analyzed using Clustal Omega. We also aligned the protein sequences of human fibrillin 1, 2, and 3 and predicted the conservation among the homologs of the fibrillin family.^18^

Using the protein structure modeling tool SWISS‐MODEL,^21^ we predicted the structure of the wild‐type and mutant for the cbEGF motifs 18‐19 (residues 1367‐1448).^18^ The structure prediction was performed using the cbEGF‐like domain pair of human fibrillin‐1 as the template (PDB:1emn). The similarity in sequences between human *FBN1* and *FBN2* was 78%.

### 3.3 Segregation analysis

Direct Sanger sequencing was carried out to identify the potential disease‐causing mutations. PCR primers were as follow: 5′‐GGAATAGCACCATCTATTATTGG‐3′ (forward) and 5′‐TTAGGCAGGAATTATCTTGCAA‐3′ (reverse).

Mutations in unrelated controls were screened using a RG‐6000 real‐time HRM system as described by us previously. Details are shown in Appendix S1.

## 4 RESULTS

After the filtering steps, a solitary heterozygous missense variant, c.4177T>G (NM_001999), in the FBN2 gene (NM_001999) was identified in three affected patients based on sequences from the OMIM and HGMD databases. The mutation was located in exon 32 and confirmed by Sanger sequencing (Figure 1C). Another rare variant, rs757406333 (c.1643A>C, p.Asp548Ala) in *FBN2*, was also observed in all three samples; however, the functional prediction indicated that this variant could be tolerated. Sanger sequencing revealed that the c.4177T>G mutation of *FBN2* was present in all individuals with the CCA phenotype, but not in any of the individuals without CCA (Figure 1C), demonstrating that the mutation cosegregated with the disease phenotype in this family. The *FBN2* c.4177T>G (p.Cys1393Gly) mutation was not present in any public genomic variants database, including 1000Genomes, ESP6500, ExAC Browser (exome aggregation consortium), and COSMIC (the catalog of somatic mutations in cancer). Additionally, the c.4177T>G mutation in *FBN2* was also not present in 500 normal unrelated controls that were screened, nor was the mutation present in the UMD‐FBN2 and HGMD databases. These results indicated that c.4177T>G in *FBN2* is a novel mutation that may cause CCA.

**Figure 2:**
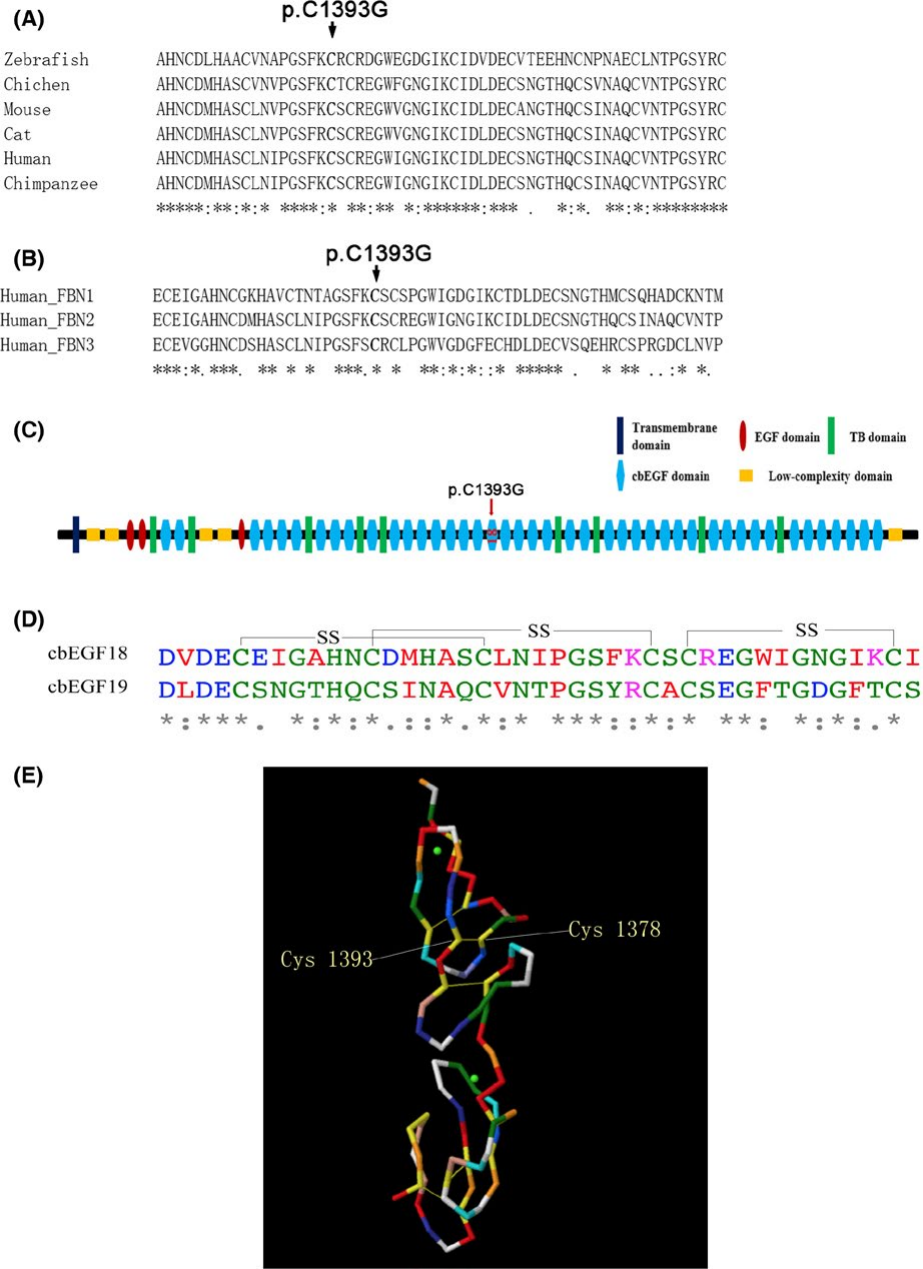
Conservation analysis of *FBN2* p.Cys1393. A, Phylogenetic comparison of FBN2 across species. B, Phylogenetic comparison of human fibrillin‐1, 2, and 3. C, The putative structural domains. The p.Cys1393Gly mutation identified was located in cbEGF domain 18. D, Multiple sequence alignment of cbEGF domain 18‐19 of fibrillin‐2. Disulfide bonds are shown indicated by black lines (SS). Location of Cys 1393 is shown by the red arrow. E, Modeled structure of cbEGF domain 18 and 19 of fibrillin‐2. The disulfide bonds are shown by green lines

The heterozygous c.4177T>G mutation results in a cysteine to glycine substitution at amino acid residue 1393. The conservation analysis via Clustal Omega revealed that cysteine 1393 of human *FBN2* is phylogenetically conserved among various species including human, chimpanzee, cat, mouse, chicken, and zebrafish (Figure 2A) and showed high conservation when compared with human *FBN1* and *FBN3* (Figure 2B). Using variant functional prediction tools, this mutation was predicted to be damaging or to have a high functional impact (SIFT, score = 0, damaging; PolyPhen‐2, score = 0.997, possible damaging; Mutation Taster, score = 159, disease causing; Mutation Assessor, score = 5.52, high functional impact; FATHMM, score = −5.9, damaging). These results indicated that the *FBN2* c.4177T>G (p.Cys1393Gly) mutation was the disease‐causing mutation.

## 5 DISCUSSION

Congenital contractural arachnodactyly is a rare autosomal dominant disorder and has been reported to have various clinical manifestations and intragenic heterogeneity.^16^, ^22^ CCA is genetically distinct from MFS. CCA has only been observed in individuals with mutations in *FBN2*; however, individuals with MFS have mutations in *FBN1*,* TGFBR2*, and *TGFBR1*.^8^, ^16^, ^22^, ^23^

*FBN2*, which encodes fibrillin‐2, contains 2,912 amino acids. Fibrillins (fibrillin‐1, 2, and 3) can form microfibrils,^24^, ^25^, ^26^ which play important roles in the structural integrity of organs. Fibrillins and microfibrils act as a scaffold in the process of elastogenesis,^26^, ^27^, ^28^ and abnormalities in fibrillins have been identified in a series of hereditary connective tissue diseases.^29^, ^30^, ^31^

Fibrillin‐2 is composed of three EGF domains, nine TGFβ‐binding protein‐like domains, and 43 cbEGF domains (Figure 2C).^18^, ^21^ The structure of the cbEGF domain of fibrillin‐2 is stabilized by six cysteine residues that form three stabilizing disulfide bonds with a 1‐3, 2‐4, and 5‐6 pattern (Figure 2D).^18^, ^21^, ^32^, ^33^, ^34^ Cysteine 1393 located within the 18th cbEGF domain of the fibrillin‐2 was predicted to interact with cysteine 1378 via a disulfide bond (Figure 2E). In the present pedigree, the c.4177T>G mutation resulted in a cysteine to glycine substitution, resulting in a loss of the disulfide bond between cysteines 1393 and 1378. The mutation may reduce the stability of the structure of fibrillin‐2 and thereby result in the symptoms of CCA.

In conclusion, through exome sequencing of a five‐generation Chinese family, we identified a novel missense mutation of *FBN2* (c.4177T>G, p.Cys1393Gly), which resulted in a cysteine to glycine substitution at residue 1393 that cosegregated with CCA. The cysteine 1393 residue is highly conserved, and the mutation results in the loss of a disulfide bond between cysteines 1393 and 1378 of fibrillin‐2. Our study demonstrates that the p.Cys1393Gly is the pathogenic mutation in this family and also a novel disease‐causing mutation of CCA.

## CONFLICT OF INTEREST

None declared.

## AUTHORSHIP

SZ, HW, and PW: designed the study. FW, PW, PZ, YD, JZ, and GH: involved in experimental and data analysis. SZ and PW: drafted the manuscript. QKW and CX: involved in critical revision of the manuscript. SZ and PW: supervised the study.

## References

[1] Philip N , Garcia‐Meric P , Wernert F . The Beals‐Hecht syndrome (congenital contractural arachnodactyly) revealed in a neonate. Pediatrie. 1988;43(7):609‐612. 3200664

[2] Epstein CJ , Graham CB , Hodgkin WE , Hecht F , Motulsky AG . Hereditary dysplasia of bone with kyphoscoliosis, contractures, and abnormally shaped ears. J Pediatr. 1968;73(3):379‐386. 5667420 10.1016/s0022-3476(68)80115-5

[3] Inbar‐Feigenberg M , Meirowitz N , Nanda D , Toi A , Okun N , Chitayat D . Beals syndrome (congenital contractural arachnodactyly): prenatal ultrasound findings and molecular analysis. Ultrasound Obstet Gynecol. 2014;44(4):486‐490. 24585410 10.1002/uog.13350

[4] Ramos Arroyo MA , Weaver DD , Beals RK . Congenital contractural arachnodactyly. Report of four additional families and review of literature. Clin Genet. 1985;27(6):570‐581. 4017278 10.1111/j.1399-0004.1985.tb02042.x

[5] Hecht F , Beals RK . “New” syndrome of congenital contractural arachnodactyly originally described by Marfan in 1896. Pediatrics. 1972;49(4):574‐579. 4552107

[6] Meinecke P . Marfan‐like features and congenital contractural arachnodactyly. J Pediatr. 1982;100(6):1006‐1007. 10.1016/s0022-3476(82)80553-2 7086582

[7] Belleh S , Zhou G , Wang M , Der Kaloustian VM , Pagon RA , Godfrey M . Two novel fibrillin‐2 mutations in congenital contractural arachnodactyly. Am J Med Genet. 2000;92(1):7‐12. 10797416

[8] Frederic MY , Monino C , Marschall C , Hamroun D , Faivre L , Jondeau G . The FBN2 gene: new mutations, locus‐specific database (Universal Mutation Database FBN2), and genotype‐phenotype correlations. Hum Mutat. 2009;30(2):181‐190. 18767143 10.1002/humu.20794

[9] Gupta PA , Putnam EA , Carmical SG , Kaitila I , Steinmann B , Child A . Ten novel FBN2 mutations in congenital contractural arachnodactyly: delineation of the molecular pathogenesis and clinical phenotype. Hum Mutat. 2002;19(1):39‐48. 11754102 10.1002/humu.10017

[10] Liu W , Zhao N , Li XF , Wang H , Sui Y , Lu YP . A novel FBN2 mutation in a Chinese family with congenital contractural arachnodactyly. FEBS Open Bio. 2015;5:163‐166. 10.1016/j.fob.2015.02.005 PMC4359973 25834781

[11] Nishimura A , Sakai H , Ikegawa S , Kitoh H , Haga N , Ishikiriyama S . FBN2, FBN1, TGFBR1, and TGFBR2 analyses in congenital contractural arachnodactyly. Am J Med Genet A. 2007;143A(7):694‐698. 17345643 10.1002/ajmg.a.31639

[12] Park ES , Putnam EA , Chitayat D , Child A , Milewicz DM . Clustering of FBN2 mutations in patients with congenital contractural arachnodactyly indicates an important role of the domains encoded by exons 24 through 34 during human development. Am J Med Genet. 1998;78(4):350‐355. 9714438

[13] Putnam EA , Zhang H , Ramirez F , Milewicz DM . Fibrillin‐2 (FBN2) mutations result in the Marfan‐like disorder, congenital contractural arachnodactyly. Nat Genet. 1995;11(4):456‐458. 7493032 10.1038/ng1295-456

[14] Sengle G , Carlberg V , Tufa SF , Charbonneau NL , Smaldone S , Carlson EJ . Abnormal activation of BMP signaling causes myopathy in Fbn2 null mice. PLoS Genet. 2015;11(6):e1005340. 26114882 10.1371/journal.pgen.1005340 PMC4482570

[15] Yagi H , Hatano M , Takeda N , Harada S , Suzuki Y , Taniguchi Y . Congenital contractural arachnodactyly without FBN1 or FBN2 gene mutations complicated by dilated cardiomyopathy. Intern Med. 2015;54(10):1237‐1241. 25986263 10.2169/internalmedicine.54.4280

[16] Guo X , Song C , Shi Y , Li H , Meng W , Yuan Q . Whole exome sequencing identifies a novel missense FBN2 mutation co‐segregating in a four‐generation Chinese family with congenital contractural arachnodactyly. BMC Med Genet. 2016;17(1):91. 27912749 10.1186/s12881-016-0355-6 PMC5135809

[17] Lavillaureix A , Heide S , Chantot‐Bastaraud S , Marey I , Keren B , Grigorescu R . Mosaic intragenic deletion of FBN2 and severe congenital contractural arachnodactyly. Clin Genet. 2017;92(5):556‐558. 28762477 10.1111/cge.13062

[18] You G , Zu B , Wang B , Wang Z , Xu Y , Fu Q . Exome sequencing identified a novel FBN2 mutation in a Chinese family with congenital contractural arachnodactyly. Int J Mol Sci. 2017;18(4):E626. 28379158 10.3390/ijms18040626 PMC5412266

[19] Davis MR , Summers KM . Structure and function of the mammalian fibrillin gene family: implications for human connective tissue diseases. Mol Genet Metab. 2012;107(4):635‐647. 22921888 10.1016/j.ymgme.2012.07.023

[20] Chaudhry SS , Gazzard J , Baldock C , Dixon J , Rock MJ , Skinner GC . Mutation of the gene encoding fibrillin‐2 results in syndactyly in mice. Hum Mol Genet. 2001;10(8):835‐843. 11285249 10.1093/hmg/10.8.835

[21] Piha‐Gossack A , Sossin W , Reinhardt DP . The evolution of extracellular fibrillins and their functional domains. PLoS ONE. 2012;7(3):e33560. 22438950 10.1371/journal.pone.0033560 PMC3306419

[22] Maslen C , Babcock D , Raghunath M , Steinmann B . A rare branch‐point mutation is associated with missplicing of fibrillin‐2 in a large family with congenital contractural arachnodactyly. Am J Hum Genet. 1997;60(6):1389‐1398. 9199560 10.1086/515472 PMC1716103

[23] Babcock D , Gasner C , Francke U , Maslen C . A single mutation that results in an Asp to His substitution and partial exon skipping in a family with congenital contractural arachnodactyly. Hum Genet. 1998;103(1):22‐28. 9737771 10.1007/s004390050777

[24] Charbonneau NL , Carlson EJ , Tufa S , Sengle G , Manalo EC , Carlberg VM . In vivo studies of mutant fibrillin‐1 microfibrils. J Biol Chem. 2010;285(32):24943‐24955. 20529844 10.1074/jbc.M110.130021 PMC2915730

[25] Charbonneau NL , Jordan CD , Keene DR , Lee‐Arteaga S , Dietz HC , Rifkin DB . Microfibril structure masks fibrillin‐2 in postnatal tissues. J Biol Chem. 2010;285(26):20242‐20251. 20404337 10.1074/jbc.M109.087031 PMC2888437

[26] Piha‐Gossack A , Sossin W , Reinhardt DP . The evolution of extracellular fibrillins and their functional domains. PLoS ONE. 2012;7(3):e33560. 22438950 10.1371/journal.pone.0033560 PMC3306419

[27] Jacobson SL , Kimberly D , Thornburg K , Maslen C . Localization of fibrillin‐1 in the human term placenta. J Soc Gynecol Investig. 1995;2(5):686‐690. 10.1016/1071-5576(95)97405-6 9420876

[28] Reinhardt DP , Sasaki T , Dzamba BJ , Keene DR , Chu ML , Gohring W . Fibrillin‐1 and fibulin‐2 interact and are colocalized in some tissues. J Biol Chem. 1996;271(32):19489‐19496. 8702639 10.1074/jbc.271.32.19489

[29] Li J , Wu W , Lu C , Liu Y , Wang R , Si N . Gross deletions in FBN1 results in variable phenotypes of Marfan syndrome. Clin Chim Acta. 2017;474:54‐59. 28842177 10.1016/j.cca.2017.08.023

[30] Li Y , Xu J , Chen M , Du B , Li Q , Xing Q . A FBN1 mutation association with different phenotypes of Marfan syndrome in a Chinese family. Clin Chim Acta. 2016;460:102‐106. 27353645 10.1016/j.cca.2016.06.031

[31] Yang H , Luo M , Chen Q , Fu Y , Zhang J , Qian X . Genetic testing of the FBN1 gene in Chinese patients with Marfan/Marfan‐like syndrome. Clin Chim Acta. 2016;459:30‐35. 27234404 10.1016/j.cca.2016.05.021

[32] McGowan SE , Holmes AJ , Mecham RP , Ritty TM . Arg‐Gly‐Asp‐containing domains of fibrillins‐1 and ‐2 distinctly regulate lung fibroblast migration. Am J Respir Cell Mol Biol. 2008;38(4):435‐445. 18006876 10.1165/rcmb.2007-0281OC

[33] Robertson I , Jensen S , Handford P . TB domain proteins: evolutionary insights into the multifaceted roles of fibrillins and LTBPs. Biochem J. 2011;433(2):263‐276. 21175431 10.1042/BJ20101320

[34] Trask TM , Trask BC , Ritty TM , Abrams WR , Rosenbloom J , Mecham RP . Interaction of tropoelastin with the amino‐terminal domains of fibrillin‐1 and fibrillin‐2 suggests a role for the fibrillins in elastic fiber assembly. J Biol Chem. 2000;275(32):24400‐24406. 10825173 10.1074/jbc.M003665200

