# Supplementary material for: A novel FBN2 mutation cosegregates with congenital contractural arachnodactyly in a five‐generation Chinese family
Source: Clin Case Rep. 2018 Jul 3;6(8):1612–7. doi: 10.1002/ccr3.1693 (PMC6099051; doi:10.1002/ccr3.1693)
Supplement: Supplementary file 1 [file CCR3-6-1612-s001.docx]

**Supplemental Information**

**Supplemental Methods**

1. **Whole exome sequencing**

The reads of each individual were aligned to the human genome reference (hg19) using BWA aligner, PCR duplicates were marked using Mark Duplicates program in Picard tool set. GATK and Samtools were used for the identification of insertion-deletion polymorphisms (INDELs) and the single nucleotide variants (SNVs). Additionally, standard hard filtering parameters were used to perform SNVs and INDELs discovery and genotyping. All variants were annotated using the Annovar program. All candidate mutations in the subject were filtered against the single nucleotide polymorphism databases (dbSNP build 137), ethnic Han Chinese individuals from Beijing available in the 1000 Genomes Project, HapMap, and Nonsynonymous variants (NSVs) including splicing sites and indel variants were selected.

We obtained 12.2, 12.9 and 13.3 billion bases of sequence IV:7, V:5 and V:6, respectively. After mapping to the human reference genome (hg19), we achieved 53-55% capture efficiency rate on target regions which mapped to the targeted exome region with a mean depth from 134×, 142× and 142× for IV:7, V:5 and V:6, respectively. Of these, an average of 86.91%-88.70% of the reads had Q scores greater than 30, which indicate a sufficient depth to interrogate the exons formutations.

After conducting the base quality score recalibration, indel realignment, duplicate removal, SNP and INDEL discovery and genotyping, we detected 48,000, 48, 465 and 48,300 single nucleotide variations in the exome prior to filtering, and from for IV:7, V:5 and V:6, respectively. Based on the annotation from the ANNOVAR, 20,648, 20,446 and 20,723 nonsynonymous variants (NSVs) including the SNVs, splicing sites and indel variants were found in exome of IV:7, V:5 and V:6 respectively.

For the next step, we filtered out the variants that already existed in dbSNP137, and the minor allele frequency greater than 1% according the data of 1000Genomes, since the CCA disease-causing variant should be rare in the general population. Variants did not share by all sequenced and affected individuals were excluded. Given the apparent autosomal dominant mode of inheritance in this pedigree, variants showed homozygous sequence were further excluded. After filtering, 54 NSVs were included in further analysis. We further used variant functional prediction tools, including SIFT, PolyPhen-2, Mutation Taster, Mutation Assessor, and FATHMM to predict of deleterious nonsynonymous SNVs. Finally, a solitary heterozygous missense variant, c.4177T>G (NM_001999) in Exon 32 of the FBN2 gene（NM_001999）, was identified in all three sequenced affected individuals based on the OMIM and the HGMD. The mutation was confirmed by Sanger sequencing (Fig. 3). Another rare variant, rs757406333 (c.1643A>C, p.Asp548Ala) in *FBN2* was also observed in all three samples, however, the functional prediction indicate it maybe tolerated.

1. HRM mutation screening in controls

The HRM technology is based on the different molecular physical properties of DNA molecules on the fragment length, GC content and GC distribution, which makes DNA molecules with different genotypes (with or without mutation) have different shapes and positions of its dissolution curves when heated at different temperatures [27-29]. The different genotypes for a genomic variant can then be distinguished based on their different dissolution curves. The polymerase chain reactions (PCR) for genotyping was performed in a 25 μl mixture with 2.5 μl of 10x PCR buffer, 10 mM dNTP (0.5 μl), 25 mM Mg^2+^ (1.5 μl), 5 pmol of each primer, 25 ng of genomic DNA, and 0.7 μl of 5 mM SYTO9. PCR was performed on an ABI9700 System (Applied Biosystems, Foster City, CA) with a thermal profile of 95°C for 3 minutes, 40 cycles of 95°C for 10 seconds, 61°C or other appropriate annealing temperatures for 10 seconds and 72°C for 10 seconds, and 72°C for 10 minutes. Primers for PCR of HRM were 5’-GGAAAGTGGCTGACAGTTGTTT -3’ (forward) and 5’-AGGCATGCATGTCGCAGTTAT -3’ (reverse). PCR products were directly genotyped using the HRM analysis on a Rotor-Gene 6000 System. Ten samples were randomly selected for direct Sanger sequencing to verify the accuracy of HRM genotyping.
